# Psychological well-being among older adults during the COVID-19 outbreak: a comparative study of the young–old and the old–old adults

**DOI:** 10.1017/S1041610220000964

**Published:** 2020-05-22

**Authors:** J. López, G. Perez-Rojo, C. Noriega, I. Carretero, C. Velasco, J.A. Martinez-Huertas, P. López-Frutos, L. Galarraga

**Affiliations:** 1 Department of Psychology and Pedagogy, School of Medicine, Universidad San Pablo-CEU, CEU Universities, Madrid, Spain; 2 Department of Cognitive Psychology, Universidad Autónoma de Madrid, Madrid, Spain

**Keywords:** well-being, older adults, crisis, stress, personal strengths, eudaimonia, personal growth, purpose in life

## Abstract

The COVID-19 outbreak could be considered as an uncontrollable stressful life event. Lockdown measures have provoked a disruption of daily life with a great impact over older adults’ health and well-being. Nevertheless, eudaimonic well‐being plays a protective role in confronting adverse circumstances, such as the COVID-19 situation. This study aims to assess the association between age and psychological well-being (personal growth and purpose in life). Young–old (60–70 years) and old–old (71–80 years) community-dwelling Spaniards (*N* = 878) completed a survey and reported on their sociodemographic characteristics and their levels of health, COVID-19 stress-related, appraisal, and personal resources. Old–old did not evidence poorer psychological well-being than young–old. Age has only a negative impact on personal growth. The results also suggest that the nature of the COVID-19 impact (except for the loss of a loved one) may not be as relevant for the older adults’ well-being as their appraisals and personal resources for managing COVID-related problems. In addition, these results suggest that some sociodemographic and health-related variables have an impact on older adults’ well-being. Thus, perceived-health, family functioning, resilience, gratitude, and acceptance had significant associations with both personal growth and purpose in life. Efforts to address older adults’ psychological well-being focusing on older adults’ personal resources should be considered.

## Introduction

The COVID-19 pandemic overspreads around the world and older persons are at high risk. Spain has emerged as one of the most affected countries with a high prevalence rate. Although the virus can affect everybody, older adults are a particularly vulnerable population (Verity *et al.*, 2020). Research has documented the negative consequences of COVID-19, both physically and psychologically. COVID-19 outbreak is a loss and potentially traumatic event. Although research has been dominated by a psychopathological approach, people showed heterogeneity of outcomes following a loss and adverse events (Flint *et al.*, 2020).

There is a growing awareness among researchers that well-being is not only the absence of emotional distress, but rather implies a positive physical, mental, and social condition. Psychological well-being is defined as an effort to improve ourselves and fulfill our potential, which is related with having a purpose in life and sense of life, coping with challenges and making a certain effort to overcome and achieve valuable goals. Personal growth (the extent to which individuals develop potential by growing and expanding as a person) and purpose in life (viewing one’s life as having meaning, direction, and goals) are the core components of positive psychological functioning. Personal growth and purpose in life seem to decline with age when older, young, or midlife adults are compared (Ryff, 2014, Ryff & Keyes, 1995). However, research has shown that personal strengths, like resilience and gratitude, are associated with psychological well-being in older adults and it contributes to making older people more satisfied with their lives (Ryff, 2014; Smith and Hollinger-Smith, 2015; Wood *et al.*, 2009).

Older adults have been found to be with decreased levels of personal growth and purpose in life associated with higher age. Nevertheless, available empirical data about differences in psychological well-being between young–old and old–old are limited and inconclusive in community-dwelling older adults (Meléndez *et al.*, 2018).

Lazarus and Folkman (1984) developed a particularly helpful and extensively used stress process model that proposes a series of interrelated conditions. From this perspective, primary stressors, such as direct and indirect problems associated with COVID-19 (having symptoms, being hospitalized, a loved one hospitalization or loss), create the conditions under which emotional distress may occur, but the extent to which older adults experience distress or psychological well-being depends on their appraisal style and the resources they may have to assist in managing stressors. Based on this model, the current study investigated the psychological well-being experienced by young–old and old–old adults during the COVID-19 crisis and the variables associated.

## Methods

We undertook a cross-sectional study of community-dwelling older adults living in Spain during the COVID-19 lockdown. Participants were recruited through older people’s associations and organizations from different backgrounds, social media (Twitter, WhatsApp, LinkedIn), and using the snowball sampling technique. Nine hundred and fifty-seven people completed the survey. Only one participant did not sign the informed consent and 78 were removed because of not meeting the inclusion criteria: 8 were living in a nursing home, 2 did not specify if they were living in the community, 26 were under 60 years old, and 42 did not specify their age. The study was approved by the University Ethics Committee.

A Web-based survey was conducted to collect information about sociodemographic characteristics of older adults, self-perceived health, and features of the COVID-19 lockdown situation. The following standardized questionnaires were also Web-based administrated:

- *The Family APGAR* (Smilkstein, 1978). This 5-items scale was used to measure family functioning (adaptability, partnership, growth, affection, and resolve). Items were scored with a 3-point Likert scale ranging from 0 (hardly ever) to 2 (usually). We used the Spanish version (Bellón *et al.*, 1996) which showed adequate reliability in our sample (Cronbach’s *α* = .788).
- *Brief Resilient Coping Scale (BRCS)* (Sinclair and Wallston, 2004). This 4-item scale was used to measure resilience. It was assessed with a 5-point Likert scale ranging from 1 (nothing) to 5 (a lot) in which the person was asked to indicate the degree the statement reflected the way he or she usually reacts. The Spanish version (Tomás *et al.*, 2012) showed adequate reliability in our sample (Cronbach’s *α* = .792).
- *Gratitude Subscale of the Values in Action Inventory of Strengths-Short Form* (Littman-Ovadia, 2015). This 5-item scale was used to measure gratitude. It included 5-point Likert scale response options ranging from 1 (very different to me) to 5 (very similar to me). The Spanish version (Azañedo *et al.*, 2017) showed good reliability in our sample (Cronbach’s *α* = .803).
- *The Acceptance and Action Questionnaire - II (AAQ-II)* (Bond *et al.*, 2011). This 7-items instrument was used to measure experiential avoidance and psychological inﬂexibility. Participants had to indicate the degree to which a series of thoughts and feelings described him or her, scoring from 1 (not at all true) to 7 (completely true). The Spanish version (Ruiz *et al.*, 2013) showed good reliability in our sample (Cronbach’s *α* = .893).
- *Ryff’s Psychological Well-Being Scales* (Ryff, 1989). Specifically, two subscales, personal growth (the extent to which they were making use of their talents and potential) and purpose in life (the extent to which respondents felt their lives had meaning, purpose, and direction), measured by 7 and 6 items, respectively, were used to measure psychological well-being. Both scales were scored in a 7-point Likert scale ranging from 1 (never) to 7 (always). The Spanish version (Diaz *et al.*, 2006) showed adequate reliability for personal growth (Cronbach’s *α* = .625) and purpose in life (Cronbach’s *α* = .808).

## Results

The sample was made up of 878 community-dwelling older adults from Spain (626 from 60 to 70 and 252 from 71 to 80 years old) 3 weeks following the lockdown restrictions. Older adults stay permanently at home and only go outside for an essential reason such as buying bread, food, water, or collecting medication. Most participants were women (62%), were living with their spouse or partner (63.80%), had a highly functional family (89.5%), and reported a good (39.60%) or fair (37.80%) perceived health. Moreover, 53 participants had COVID-19 symptomatology; 6 had been hospitalized, 114 had a close family member or friend who had been hospitalized, and 72 reported the loss of a loved one by the virus (Table 1). There were no signiﬁcant differences between groups (young–old and old–old) in any direct or indirect affection by COVID-19 variable.

As expected, young–old experienced more personal growth (F (1, 876) = 9.090, *p* = .003), but they didn’t experience more purpose in life (F (1, 876) = .123, *p* = .726) than old–old. No significant group differences emerged in older adults’ appraisals or COVID stress-related variables. Furthermore, both groups of older adults showed differences in gratitude and resilience. Specifically, old–old experienced more gratitude (F (1, 876) = 3.888, *p* = .049) and more resilience (F (1, 861) = 12.653, *p* = .001).

Staged stepwise regression analyses were used to explain the influence of sociodemographic and health-related, COVID-19 stress-related, appraisal, and resource variables on older adults’ personal growth and purpose in life, following the stress process model (Tables 2 and 3). In the first step, less age, better perceived health, being married or living with a partner, and more family functioning scores were related to more personal growth levels. In the second step, COVID stress-related variables, only the loss of a loved one by COVID-19 was related with personal growth. In the third step, appraisal, it was found that more fear of COVID-19 outbreak was significantly related to less personal growth levels. In the fourth step, resources, more resilience and gratitude and less experiential avoidance were significantly associated with greater levels of personal growth. In the case of purpose in life, better perceived health and family functioning were related to greater purpose in life. In the second step, regarding COVID stress-related variables, only the loss of a loved one by COVID-19 was related with purpose in life. None of the appraisal variables were significant, but all personal resources, including more resilience and gratitude and less experiential avoidance, were significantly associated with more purpose in life.

**Table 1.** Sample characteristics

	young–old adults (*N* = 626)	old–old adults (*N* = 252)
Gender (male)	117 (33.3%)	125 (43.4%)
Marital status (married)	219 (35.00%)	99 (39.30%)
Perceived health		
Very poor	0 (0.0%)	1 (0.3%)
Poor	23 (6.6%)	24 (8.3%)
Fair	123 (35.0%)	115 (39.9%)
Good	147 (41.9%)	106 (36.8%)
Very good	58 (16.5%)	42 (14.6%)
APGAR	8.69 (1.94)	8.91 (1.66)
COVID-19 consequences:		
Symptomatology	36 (10.3%)	17 (5.9%)
Hospitalization	6 (1.00%)	3 (1.20%)
Loved one hospitalization	126 (20.10%)	46 (18.30%)
Loss of a loved one	58 (9.30%)	30 (11.90%)
Fear of COVID-19 outbreak	1.51 (.86)	1.47 (.88)
BRCS	15.58 (3.19)	16.43 (3.18)
Gratitude	22.12 (2.90)	22.54 (2.87)
AAQ-II	19.74 (6.73)	18.96 (6.59)
Personal growth	28.54 (4.43)	27.55 (4.30)
Purpose in life	28.00 (4.62)	28.12 (4.93)

Data are presented as mean (SD), or *n* (%). APGAR = Family APGAR Scale (family function); BRCS = Brief Resilient Coping Scale; AAQ-II = Acceptance and Action Questionnaire (experiential avoidance); Gratitude = Gratitude subscale of the Values in Action Inventory of Strengths-Short Form; Personal growth = Ryff’s Psychological Well-Being Subscale; Purpose in life = Ryff’s Psychological Well-Being Subscale.

**Table 2.** Stepwise multiple regression equations to predict personal growth scores

		*β*	change in *R* ^2^	*F*
Block 1: SOCIODEMOGRAPHIC AND HEALTH RELATED	Age	−.225	.048	44.847**
	Perceived health	.031	.017	31.025**
	Marital status (married or living with a partner) (yes = 1)	.115	.016	26.180**
	Family functioning	−.025	.005	21.026**
				
Block 2: COVID STRESS-RELATED	Loss of a loved one by COVID-19 (1 = yes)	.119	.014	19.757**
				
Block 3: APPRAISAL	Fear of COVID-19 outbreak	−.033	.005	17.319**
				
Block 4: PERSONAL RESOURCES	Resilience	.203	.060	25.055**
	Experiential avoidance	−.138	.020	25.108**
	Gratitude	.087	.006	23.183**
Adjusted *R* ^2^		.182		

*p < .05; **p < .001.

**Table 3.** Stepwise multiple regression equations to predict purpose in life scores

		*β*	change in *R* ^2^	*F*
Block 1: SOCIODEMOGRAPHIC AND HEALTH RELATED	Family functioning	.073	.067	63.971**
	Perceived health	.057	.045	56.112**
				
Block 2: COVID STRESS-RELATED	Loss of a loved one by COVID-19 (1 = yes)	.082	.008	40.340**
			
Block 3: APPRAISAL	(No significant variables were found)			
				
Block 4: PERSONAL RESOURCES	Experiential avoidance	−.307	.154	84.043**
	Resilience	.278	.080	97.590**
	Gratitude	.146	.017	87.394**
Adjusted *R* ^2^		.370		

*p < .05; **p < .001.

## Discussion

According to Brandtstädter and Renner (1990), older people tend to apply accommodative strategies as a way of coping with new situations. The application of this type of strategies can explain the reduction of personal growth with age, since its implementation generates an adjustment of preferences and goals that, although not intentional, makes the subject adequately adapt to the new situation, reducing the perception of situational restrictions or insufficient personal resources to achieve previously valued goals.

Aging is associated with new life challenges. Some older adults experience a time of growth and personal discovery (e.g. learning new skills). Nevertheless, other older adults are centered in their losses of loved ones and their declines. The ease of transmission of COVID-19 and its potential to kill older adults (Verity *et al.*, 2020) have the potential to affect people’s psychological well-being. For some older adults, traumatic events, like the death of close family member or friend from COVID-19, can lead to growth and learning opportunities that increase their ability to weather future challenges. The impact of resilience is most evident after a traumatic event (Smith and Hollinger-Smith, 2015). History is full of ugly or unjust life examples who were nonetheless realized (Ryff and Keyes, 1995).

Older adults who are better able to savor positive experiences (i.e. the capacity to regulate the positive feelings by directing attention to positive experiences) reported more psychological well-being (Smith and Hollinger-Smith, 2015), whereas suppression, an emotion regulation strategy, is a negative predictor of well-being (Ryff, 2014). Therefore, it is not surprising that higher experiential avoidance (i.e. the unwillingness to experience unwanted thoughts, emotions, or bodily sensations) was related in our study with less psychological well-being.

In line with Wood *et al.* (2009), gratitude is related to psychological well-being because it is the keystone of positive trait, appreciating the positive in life, having more positive account of their environments, and developing productive coping strategies. Gratitude is related to a life that is meaningful and eudemonic.

Family functioning and being married are quite related to psychological well-being because it is associated with ties to others (social contacts and social support) (Ryff and Keyes, 1995). Moreover, relational harmony (i.e. the absence of relational strain) predicted higher well-being; marriage had well-being benefits; and being divorced and never married were negatively associated with well-being (Ryff, 2014). Finally, this study also supports previous research which has suggested that multiple aspects of physical functioning were relevant for psychological well-being (Ryff, 2014).

There are some limitations to be acknowledged. First, this study has a non-probability sampling that future research should try to overcome, obtaining longitudinal data to facilitate the generalization of the results. Second, we only included two dimensions from Ryff’s psychological well-being scales (purpose in life and personal growth). Nonetheless, the inclusion of these two subscales has been done before because they have been identified as the core components of psychological functioning and widely recognized as integral components of eudaimonia (Streib *et al.,* 2010). Third, these findings are limited to community-dwelling older-adults, without severe cognitive impairment and who were able and willing to complete the survey. Consequently, the results likely underestimate the impact of cognitive impairment or long-term facilities on Spanish older adults’ well-being. Further research is needed to assess well-being in older adults with severe cognitive impairment, and living in nursing homes or other long-term care facilities. Finally, data were self-reported, and this can have increased participants’ social desirability. Future studies could include other reliable measures, such as participants’ objective health features (accompanying diseases affecting somatic or neuropsychiatric functioning, concomitant medication, etc.) provided by healthcare professionals.

Despite these limitations, our results do not show evidence that old–old have poorer psychological well-being than young–old. Age has only a significant impact on personal growth. The results also suggest that the nature of the COVID-19 impact (except for the loss of a loved one) may not be as important for the older adults’ well-being as their appraisals and personal resources for managing COVID-related problems. Some sociodemographic and health-related variables also have shown an impact on older adults’ well-being. Therefore, perceived-health, family functioning, resilience, gratitude, and acceptance had significant associations with both personal growth and purpose in life.

Although some traumatic events are highly prevalent stressors among older adults, many individuals report high levels of psychological well-being. It is necessary to develop resources such as resilience, gratitude and acceptance, family functioning, and perceived health to improve psychological well-being, especially on crisis times.
